# Access to healthcare services for migrant and left-behind children: A scoping review

**DOI:** 10.1016/j.jmh.2026.100425

**Published:** 2026-07-01

**Authors:** Jane Spiteri, Anne Carolina Ramos, Catarina Pinheiro Mota, Egemen İpek, Viorela Ducu, Bárbara Bäckström

**Affiliations:** a Department of Early Childhood & Primary Education, Faculty of Education, University of Malta, Msida, Malta; b Department of Educational Sciences, Faculty of Educational Sciences, University of Fribourg, Fribourg, Switzerland; c University of Trás-os-Montes and Alto Douro, Vila Real, Portugal; d Center of Psychology at University of Porto, Porto, Portugal; e Department of Finance & Banking, Faculty of Applied Science, Tarsus University, Mersin Province, Turkey; f Centre for the Study of Transnational Families, Faculty of Political, Administrative and Communication Sciences and Faculty of Sociology and Social Work, Babeș-Bolyai University, Cluj-Napoca, Romania; g Lead/Universidade Aberta, Lisbon, Portugal; h Rise Health, University of Trás os Montes and Alto Douro (UTAD), Vila Real, Portugal; i Lead/Universidade Aberta, Lisbon, Portugal and Centre for Social Sciences _ Cics.Nova/UNL, Lisbon, Portugal; j Rise Health UTAD, Vila Real, Portugal; k Interdisciplinary Centre of Social Sciences (CICS.NOVA), Lisbon, Portugal

**Keywords:** Migrant children, Left-behind children, Healthcare access, Child health equity, Migration and health, Scoping review, Health systems

## Abstract

Migrant and left-behind children experience health vulnerability that are shaped by climate change, including by climate change, including illness, displacement, and family separation, but also by the organisation of healthcare systems. This scoping review maps the evidence on healthcare services for migrant and left-behind children, with attention to health needs, service provision, barriers and facilitators to access, and gaps in care. Following PRISMA-ScR reporting guidance, searches were conducted in Web of Science/MEDLINE and Scopus for peer-reviewed English-language publications from the database inception to May 2026. After duplicate removal and two-stage screening, 35 publications were included. The evidence shows that migrant and left-behind children require a continuum of preventive, primary, specialist, developmental, nutritional, dental, mental health, and emergency care. However, access is frequently constrained by legal exclusion, documentation requirements, cost, language barriers, limited health system navigation, discrimination, mobility, disrupted continuity of care, and under-resourced services. Left-behind children remain especially under-represented, with limited evidence on preventive, mental health, and chronic care access. Facilitators include inclusive entitlements, community outreach, school provision, interpreters, bilingual professionals, culturally responsive care, health literacy support, and cross-sector coordination. The review argues that healthcare inequity for children affected by migration is produced not only by service absence, but by systems that fail to adapt to children’s legal, linguistic, relational, and transnational realities. Rights-based child health policy should therefore move from episodic and discretionary provision towards universal entitlements, continuous care pathways, and integrated support that is culturally responsive, developmentally appropriate, and accountable to the lived experiences of migrant and left-behind children.

## 1 Introduction

Children’s access to healthcare is both a public health priority and a child-rights obligation. Article 24 of the United Nations Convention on the Rights of the Child recognises every child’s right to the highest attainable standard of health and to facilities for the treatment of illness and rehabilitation of health (United Nations, 1989). This right is especially significant for children affected by migration, whose health needs may be intensified by displacement, poverty, interrupted care, language barriers, uncertain legal status, family separation, and exclusion from welfare systems (Abubakar et al., 2018; Murphy et al., 2020; Woodward et al., 2014). Yet formal rights do not automatically translate into meaningful access. Across countries, children’s eligibility for healthcare often remains mediated by migration status, documentation, insurance rules, local administrative practice, and the ability of families or caregivers to navigate unfamiliar systems (Østergaard et al., 2017; Palm et al., 2017).

This scoping review maps and synthesises evidence on access to healthcare services for migrant and left-behind children, focusing on health needs, barriers and facilitators to access, and gaps in service provision across different contexts. In this review, ‘healthcare services’ are understood broadly as preventive, promotive, curative, rehabilitative, and mental health services provided through formal health systems and related community or non-governmental structures (World Health Organization [WHO], 2023). ‘Access’ is conceptualised not simply as the availability of services, but as the interaction between healthcare systems and people’s capacity to identify need, seek care, reach services, pay for care, and engage with providers (Levesque et al., 2013). This understanding is particularly relevant for migrant and left-behind children because access to care is often shaped by intersecting dimensions of legal entitlement, affordability, approachability, acceptability, availability, cultural responsiveness, and continuity of care (Cu et al., 2021; Levesque et al., 2013).

The term ‘migrant children’ is used here as an umbrella category for children involved in migration processes. It includes children who have moved across or within national borders, such as refugee children, asylum-seeking children, undocumented migrant children, unaccompanied minors, children in transit, children of migrant farmworkers, internally displaced children, and children migrating with their families (International Organization for Migration [IOM], 2019; United Nations High Commissioner for Refugees [UNHCR], 2024). ‘Left-behind children’ are children who remain in a place of origin while one or both parents migrate elsewhere for employment or livelihood reasons, resulting in prolonged separation from parental care and altered caregiving arrangements (Fellmeth et al., 2018; Hossain et al., 2026). These populations are not identical. Children who migrate may face risks related to border regimes, detention, displacement, interrupted schooling, language barriers, unfamiliar health systems, or precarious legal status. Left-behind children may face different risks linked to parental absence, caregiving instability, remittance dependence, emotional distress, and disrupted healthcare decision-making. Nevertheless, both groups may be affected by migration-related disruptions to care, social protection, and health-system inclusion.

Existing evidence shows that migrant children may experience a wide range of health concerns, including incomplete vaccination, infectious disease, respiratory and gastrointestinal illness, malnutrition, dental problems, vision problems, developmental delay, chronic conditions, and mental health needs (Baris et al., 2022; Charania et al., 2020; Guan et al., 2018; Hjern et al., 1991; Kusuma et al., 2010; Markkula et al., 2018). Refugee, asylum-seeking, and unaccompanied children may also have heightened exposure to traumatic events, violence, insecurity, and post-migration stressors, which can contribute to anxiety, depression, post-traumatic stress symptoms, and behavioural difficulties (Barghadouch et al., 2016; Poyraz Fındık et al., 2021; Rosenberg et al., 2022). For left-behind children, systematic evidence indicates associations between parental migration and risks to mental health, nutrition, injury, and health-related behaviours, although findings remain uneven across regions and outcomes (Fellmeth et al., 2018; Guan et al., 2018; Hossain et al., 2026).

Despite this growing body of research, the literature on healthcare services for migrant and left-behind children remains fragmented. Some studies focus on specific services, such as vaccination, paediatric screening, emergency care, congenital heart disease, psychiatric care, vision problems, or dental services, while others examine barriers to access, healthcare entitlement, or caregiver experiences (Connor et al., 2007; Guan et al., 2018; Mostafa et al., 2021; Ruiz-Casares et al., 2013; Sandahl et al., 2013). Much less is known about how these findings connect across migrant categories, regions, and forms of healthcare provision. Left-behind children are particularly under-represented in healthcare access research, despite evidence that parental migration can alter care-seeking, nutrition, mental health, and healthcare decision-making (Fellmeth et al., 2018; Guan et al., 2018; Hossain et al., 2026).

This review addresses this gap by mapping the scope and nature of evidence on healthcare services for migrant and left-behind children. Specifically, it examines: (1) the health issues affecting these children; (2) the barriers and facilitators shaping their access to healthcare; and (3) the gaps in service provision reported in the literature. By bringing these strands of evidence together, the review contributes to a more integrated understanding of healthcare inequity as a structural, relational, and rights-based issue affecting children whose lives are shaped directly or indirectly by migration.

## 2 Methods

Access to healthcare services for migrant and left-behind children is an emerging and heterogeneous field of research. A scoping review methodology was therefore selected to map the scope, nature, and extent of the available evidence (Peters et al., 2024). Scoping reviews are particularly appropriate for fields characterised by conceptual ambiguity, diverse study designs, and uneven or emerging evidence bases (Arksey and O’Malley, 2005; Levac et al., 2010). Rather than appraising the quality of individual studies, this approach supports the identification of key concepts, evidence gaps, and patterns across a broad body of literature (Peters et al., 2024).

The review was informed by the methodological framework developed by Arksey and O’Malley (2005), refined by Levac et al. (2010), and aligned with recent Joanna Briggs Institute guidance for scoping reviews (Peters et al., 2024). The review followed five stages: (1) identifying the research questions; (2) identifying relevant studies; (3) selecting studies; (4) charting the data; and (5) collating, summarising, and reporting the results.

### 2.1 Stage 1. Identifying the research questions

An initial exploratory search was undertaken to determine the scope, breadth, and nature of the literature on healthcare services for migrant and left-behind children. This preliminary work informed the development of the review protocol, eligibility criteria, search strategy, and research questions. Consistent with scoping review guidance, the questions were designed to map the available evidence, identify key concepts, and highlight gaps in knowledge, rather than evaluate intervention effectiveness (Arksey and O’Malley, 2005; Peters et al., 2024).

The review was guided by the following research questions:

1. What health issues affect migrant and left-behind children?
2. What are the barriers and facilitators to accessing healthcare for migrant and left-behind children?
3. What gaps in healthcare service provision are identified in the literature?

The search strategy was designed to retrieve studies located at the intersection of migration-related child populations and healthcare services. It incorporated three conceptual domains: (a) migration-related child populations, (b) healthcare services and healthcare access, and (c) healthcare utilisation, barriers, facilitators, and service provision. Because terminology relating to child migration varies across policy, legal, and disciplinary contexts, the search included multiple overlapping descriptors, including migrant children, refugee children, asylum-seeking children, undocumented children, displaced children, unaccompanied minors, and left-behind children. This broad approach was adopted to maximise sensitivity and reduce the risk of excluding relevant evidence that used different migration-related terminology (Peters et al., 2024).

### 2.2 Stage 2. Identifying relevant studies

A comprehensive literature search was undertaken using Web of Science Core Collection and Scopus, with MEDLINE-indexed records available through Web of Science also included. These databases were selected because they provide broad multidisciplinary coverage across public health, medicine, migration studies, social sciences, child welfare, and policy research. The combined use of Web of Science and Scopus has been recommended in evidence synthesis because it can improve multidisciplinary coverage while reducing the risk of database-specific bias (Gusenbauer and Haddaway, 2020).

The search strategy combined population terms with healthcare service-related terms using Boolean operators. Truncation symbols were used where appropriate to capture variations in terminology. The strategy was refined through pilot searches and review-team discussions to ensure adequate coverage of the review questions. The search strategy is summarised in Table 1.

**Table 1:** Search query used for the Databases.

Database	Date of search	Query	Number of publications retrieved
Web of Science/Medline	May 20, 2026	TS=("healthcare services" OR "health care services") AND TS=("refugee child*" OR "unaccompanied minor*" OR "migrant child*" OR "undocumented child*" OR "left-behind child*" OR "left behind child*" OR "displaced child*")	70
Scopus	May 20, 2026	TITLE-ABS-KEY ("healthcare services" OR "health care services") AND TITLE-ABS-KEY ("refugee child*" OR "unaccompanied minor*" OR "migrant child*" OR "undocumented child*" OR "left-behind child*" OR "left behind child*" OR "displaced child*" OR "Migrant child*")	64

The search strategy was aligned with the review questions by combining terms for migration-related child populations with terms for healthcare services. Relevance to healthcare access, utilisation, barriers, facilitators, and service gaps was then assessed during screening and data charting. This approach supported the identification of studies addressing health conditions and service provision, as well as studies reporting structural, legal, economic, cultural, and organisational influences on healthcare access. It is consistent with recommendations that scoping review searches should prioritise sensitivity and conceptual breadth when mapping complex and heterogeneous fields (Peters et al., 2024; Tricco et al., 2018).

Although specialist databases such as PubMed, Embase, CINAHL, and PsycINFO may have identified additional studies, Web of Science/MEDLINE and Scopus were selected because of their broad interdisciplinary coverage and their ability to capture literature spanning healthcare, migration, social policy, and child welfare domains. Nevertheless, the exclusion of additional specialist databases may have resulted in the omission of some relevant studies, particularly those indexed exclusively in discipline-specific databases. This limitation should be considered when interpreting the findings and points to the need for future reviews to incorporate additional databases and grey literature sources where feasible.

### 2.3 Stage 3. Study selection

The review included peer-reviewed articles written in English and published up to May 2026 that focused on healthcare services for migrant or left-behind children under the age of 18. Study selection was conducted in two sequential stages: title and abstract screening, followed by full-text review. The process was guided by predefined inclusion and exclusion criteria developed during the protocol stage and refined through team discussion.

All retrieved records were imported into a shared screening database, and duplicate records were removed. The eligibility criteria are presented in Table 2.

**Table 2:** Eligibility criteria used for study selection.

**Category**	**Inclusion criteria**	**Exclusion criteria**
Population	Children under 18 affected directly or indirectly by migration, including migrant, refugee, asylum-seeking, undocumented, internally displaced, unaccompanied, in-transit, migrant labourer/farmworker, internal migrant, and left-behind children.	Adult-only studies; studies where child-specific findings could not be separated.
Concept	Healthcare services, healthcare access, utilisation, experiences, barriers, facilitators, service gaps, and health-system responses.	Studies without a focus on healthcare service, healthcare access, or healthcare utilisation.
Context	Any country, region, or migration setting.	No geographical exclusions.
Evidence type	Peer-reviewed English-language publications indexed in the selected databases.	Conference abstracts, dissertations, book chapters, non-peer-reviewed reports, and opinion pieces.
Time period	Database inception to May 2026.	Publications outside the search period.

Studies were eligible if they focused on children younger than 18 years who were identified as migrants, refugees, asylum seekers, undocumented migrants, internally displaced children, unaccompanied minors, children in transit, children of migrant labourers or farmworkers, internal migrant children, or left-behind children. Studies focusing exclusively on adults were excluded. Studies involving mixed populations were retained only where child-specific findings could be extracted separately.

To enhance consistency, all members of the review team first screened a subset of records independently as a calibration exercise. This helped establish a shared understanding of the eligibility criteria and supported consistent interpretation of migration-related terminology across disciplinary and geographical contexts. Following calibration, each title and abstract was screened independently by two reviewers. Records that met the inclusion criteria, or whose eligibility could not be determined from the title and abstract alone, proceeded to full-text review.

Full texts were assessed against the same eligibility criteria, with particular attention to the population studied, the focus on healthcare services or healthcare access, and the relevance of the findings to the review questions. Reasons for exclusion at the full-text stage were recorded systematically. Studies were excluded if they did not focus on healthcare services, did not include migrant or left-behind children as a primary or extractable population, or did not provide findings relevant to the review objectives.

**Fig. 1:**
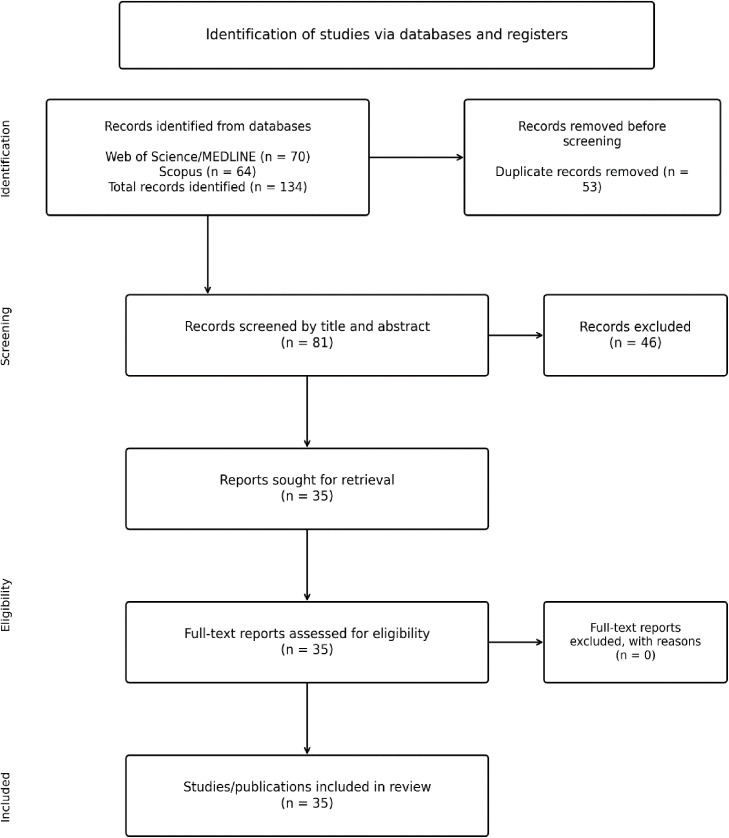
PRISMA flow diagram for the study selection process.

The review team met on six occasions during the screening process to discuss eligibility decisions, refine shared interpretations, and resolve uncertainties. Disagreements were resolved through discussion and consensus, with a third reviewer consulted where necessary. Where uncertainty remained, the article was retained for further review until agreement was reached. This collaborative process was important because terminology relating to migration, displacement, and left-behind children varies substantially across disciplines, countries, and policy contexts. The involvement of multiple reviewers helped reduce the risk of excluding relevant studies because of differences in interpretation and enhanced the transparency and trustworthiness of the selection process (Levac et al., 2010; Peters et al., 2024) (Fig. 1).

### 2.4 Stage 4. Charting the data

Data were charted using a structured data-charting form developed by the review team. The form was informed by the review questions, the PRISMA-ScR checklist, and Joanna Briggs Institute guidance for scoping reviews (Peters et al., 2024; Tricco et al., 2018). It included the following categories: author(s), year of publication, country or setting, study aim, study design, population characteristics, migration category, type of healthcare service, health issues reported, barriers to healthcare access, facilitators to healthcare access, gaps in service provision, and key findings.

A calibration exercise was undertaken before full data extraction. All authors piloted the data-charting form on a purposive subset of articles selected to reflect variation in population group, study design, and healthcare focus. This exercise assessed whether the extraction categories were sufficiently clear, consistently interpreted, and capable of capturing information relevant to the review questions.

Following the pilot extraction, the review team compared completed data-charting forms and discussed discrepancies in interpretation. Discrepancies mainly concerned the categorisation of healthcare barriers, facilitators, and service gaps, particularly where studies used overlapping concepts such as legal exclusion, financial barriers, healthcare entitlement, and service availability. These discrepancies were resolved through discussion and consensus, and the extraction form was refined to include clearer definitions and decision rules for recurring categories.

Formal inter-reviewer agreement statistics, such as Cohen’s kappa, were not calculated because the data-charting process in this scoping review was descriptive and iterative rather than designed as a formal reliability-tested coding exercise. This is consistent with scoping review methodology, where charting is often refined as familiarity with the evidence base develops (Levac et al., 2010; Peters et al., 2024). Nevertheless, several safeguards were used to enhance consistency and reliability, including piloting the extraction tool, independent extraction during calibration, team comparison of extracted data, consensus-based resolution of discrepancies, refinement of the charting form, and regular review meetings to discuss ambiguous cases.

The final data-charting form was used to produce a descriptive summary of the included literature. The characteristics of selected studies, including year of publication, country or setting, study design, population, and migration category, are presented in Appendix Table A1.

### 2.5 Stage 5. Collating, summarising, and reporting results

The extracted data were synthesised using reflexive thematic analysis to identify recurring patterns, themes, and gaps across the included studies (Braun and Clarke, 2006). Thematic analysis was selected because it provides a flexible and systematic method for synthesising diverse forms of evidence and is commonly used in scoping reviews examining complex social and healthcare phenomena (Booth et al., 2022).

The analysis proceeded through several iterative stages. First, all authors familiarised themselves with the extracted data through repeated reading of the completed data-charting forms. Second, initial codes were generated to capture key issues relating to health conditions, barriers to healthcare access, facilitators of healthcare access, and gaps in service provision. Third, related codes were grouped into broader categories and candidate themes. Fourth, themes were reviewed, refined, and discussed during team meetings to ensure that they reflected the evidence and addressed the review questions. Finally, the themes were organised into a narrative synthesis that mapped the scope of the evidence base and highlighted recurring patterns across studies.

In addition to narrative synthesis, an evidence gap map was developed to display the distribution of evidence across migration categories and healthcare service types. The map was not intended as a quality appraisal, but as a descriptive tool to identify areas of relative concentration and under-representation in the evidence base.

Consistent with scoping review methodology, the purpose of the synthesis was not to evaluate intervention effectiveness or assess methodological quality, but to map the nature, extent, and characteristics of the available evidence and identify knowledge gaps requiring further investigation (Arksey and O’Malley, 2005; Peters et al., 2024). To enhance the credibility and trustworthiness of the synthesis, preliminary findings were discussed with an independent expert in migrant and child health research who was not involved in the review process. This consultation functioned as a form of peer debriefing, allowing the review team to reflect critically on the interpretation of findings and the coherence of the thematic structure. Feedback from this consultation informed minor refinements to the interpretation of results but did not alter the underlying themes identified in the analysis.

#### 2.5.1 Protocol and registration

A scoping review protocol was developed before the review commenced to guide the formulation of the research questions, eligibility criteria, search strategy, study selection procedures, and data-charting process. The protocol was not formally registered in an open-access repository such as the Open Science Framework. Although protocol registration is increasingly encouraged to enhance transparency and reduce duplication, it is not currently a mandatory requirement for conducting or publishing scoping reviews (Peters et al., 2024; Tricco et al., 2018). In this review, the protocol functioned as an internal methodological guide and was refined iteratively as familiarity with the evidence base developed, consistent with the flexible and iterative nature of scoping review methodology. Nevertheless, the absence of prospective protocol registration is acknowledged as a limitation, as registration may have further strengthened the transparency and reproducibility of the review process.

## 3 Results

### 3.1 Overview of included publications

The review included 35 studies addressing healthcare services, healthcare access, service utilisation, barriers, facilitators, and service gaps affecting migrant and left-behind children. The publications of the included studies are summarised in Appendix Table A1.

The included literature was geographically diverse. Studies focused on countries and settings in Africa (Ethiopia, South Africa), Asia (China, India, Jordan, Lebanon, Malaysia, Singapore, Thailand, Türkiye, Yemen), Europe (Albania, Bosnia and Herzegovina, Croatia, Kosovo, North Macedonia, Portugal, Sweden, Denmark, Finland, Norway, Iceland, United Kingdom), North America (Canada, Mexico, United States), Latin America and the Caribbean (Argentina, Chile, Colombia, Dominican Republic, Nicaragua, Peru), Oceania (Australia, New Zealand), and the Darién Gap transit corridor. However, this distribution was uneven. A substantial proportion of studies came from high-income destination countries in Europe, North America, and Oceania, while fewer examined healthcare access in low- and middle-income countries, transit contexts, conflict-affected settings, or countries of origin.

The included studies also varied methodologically, comprising quantitative observational studies, cross-sectional surveys, retrospective clinical studies, qualitative studies, mixed-methods studies, systematic, narrative or scoping reviews, and policy analyses. The evidence therefore provides a broad map of the field rather than a uniform body of comparable intervention or outcome data. Study populations included international migrant children, undocumented migrant children, refugee and asylum-seeking children, children of migrant farmworkers, unaccompanied minors, internally displaced children, children in transit, children from migrant labourer settlements, and left-behind children. Refugee and international migrant children were the most frequently represented groups, while only two papers focused on left-behind children (Guan et al., 2018; Hossain et al., 2026). This indicates that the literature remains more strongly oriented towards children who relocate across or within borders than towards children who remain in origin communities while parents migrate.

### 3.2 Health issues affecting migrant and left-behind children

The first research question asked what health issues affect migrant and left-behind children. Across the included publications, health needs were multidimensional, spanning preventive, physical, developmental, nutritional, dental, reproductive, and mental health domains.

Preventive healthcare, particularly vaccination, emerged as a recurrent concern. Studies in China and India reported lower or incomplete immunisation among socioeconomically disadvantaged migrant children, especially recent migrants and children living in mobile or precarious labour contexts (Dutta et al., 2017; Hu et al., 2013; Kusuma et al., 2010). Similar concerns were identified in Latin America and the Caribbean, where migrant children often arrived without complete immunisation schedules (Calderon et al., 2024). Evidence from New Zealand showed underuse of preventive services such as immunisation (Akhtar et al., 2022), while hospitalisations for vaccine-preventable diseases varied by migrant background and visa category, with children from refugee backgrounds experiencing disproportionate burdens (Charania et al., 2020). Adolescents may also require sexual and reproductive preventive healthcare, including screening and vaccination for sexually transmitted infections, human papillomavirus, pregnancy care, screening, and condom access (Calderon et al., 2024; Kovar et al., 2021). Transit contexts also revealed risks linked to incomplete immunisation, exposure to infectious disease, and limited access to screening or follow-up (Naranjo et al., 2023).

Physical health needs included infectious diseases, such as hepatitis B, congenital syphilis, tuberculosis, and parasitic infections, as well as respiratory conditions, gastrointestinal illness, diarrhoea, wasting, dermatological problems, dental needs, inborn errors of metabolism, and chronic or specialist conditions (Ambika et al., 2026; Baris et al., 2022; Calderon et al., 2024; Ibrahim et al., 2003). Newly resettled refugee children in Sweden required nutritional assessment, dental care, and follow-up for chronic conditions, with some experiencing iron deficiency or obesity (Hjern et al., 1991). Syrian refugee children with congenital heart disease in Lebanon often presented late for tertiary cardiac care and experienced poorer surgical outcomes, illustrating how delayed access can worsen otherwise treatable conditions (Mostafa et al., 2021). Refugee children in Türkiye had higher rates of hospitalisation than resident children, particularly in neonatal and intensive care units (Baris et al., 2022), while those suspected of inherited metabolic disorders faced challenges in obtaining early diagnosis and long-term specialist management (Yoldaş Çelik and Köşeci, 2026). Environmental and occupational exposures also shaped health risks, with refugee children exposed to extreme weather conditions (Fernandes et al., 2015; Kemei et al., 2023) and children of migrant farmworkers facing increased risk of illness and injury due to pesticide exposure and hazardous working environments (Committee on Community Health Services, 2005).

Developmental and early childhood concerns were also evident. Syrian preschool refugee children living under temporary protection in Türkiye were more likely to experience developmental delays than Turkish children, underscoring the importance of developmental screening, early intervention, and linguistically accessible follow-up (Ayas et al., 2022). Children from farmworker families in the United States also presented with delayed development (Committee on Community Health Services, 2005). Among migrant labourer settlements in India, under-five children experienced diarrhoea, acute respiratory infection, and wasting, while access was shaped by health-centre timing, language barriers, and indirect costs (Ambika et al., 2026). Migrant children also faced higher risks of stillbirth, neonatal death, premature delivery, caries, and untreated dental problems (Calderon et al., 2024; Markkula et al., 2018). For left-behind children, available evidence pointed particularly to undernutrition, including wasting, underweight, and stunting linked to maternal migration, with potential implications for physical and cognitive development (Hossain et al., 2026).

Mental health was a major but unevenly serviced area. Refugee, asylum-seeking, internally displaced, and unaccompanied children were reported to experience trauma exposure, anxiety associated with migration, inadequate language skills, cultural shock, separation from support systems, depression, loneliness, post-traumatic stress symptoms, behavioural concerns, and insecurity (Alshamary et al., 2024; Barghadouch et al., 2016; Committee on Community Health Services, 2005; Misra et al., 2022; Poyraz Fındık et al., 2021; Rosenberg et al., 2022). Refugee children and adolescents were also reported to experience severe psychosocial problems, including panic attacks, sleep disorders, psychiatric comorbidity, and suicidality (Barghadouch et al., 2019; Fayad et al., 2024; Poyraz Fındık et al., 2021; Sandahl et al., 2013). Gendered risks were also noted, particularly girls’ exposure to sexual violence and associated pregnancy and psychological consequences (Kemei et al., 2023). Importantly, lower service utilisation should not be interpreted as lower need. Afghan refugee children in the United States, for example, were at risk of mental health problems but did not always receive timely diagnosis or care (Rosenberg et al., 2022). In Denmark, refugee children had fewer contacts with psychiatric healthcare services than Danish-born peers, suggesting possible under-recognition, referral barriers, or difficulties engaging with services (Barghadouch et al., 2016).

Health vulnerabilities were often embedded within wider patterns of social insecurity. Children experiencing difficulties in accessing healthcare frequently also faced challenges related to food security, housing conditions, education, personal safety, and psychological well-being, as reported among internally displaced children in Ethiopia (Kemei et al., 2023). These findings indicate that healthcare needs are closely connected to wider social determinants of child health. They also point to the importance of cross-border coordination for children whose health trajectories extend across national systems, particularly in relation to early detection and continuity of care (Yoldaş Çelik and Köşeci, 2026).

### 3.3 Barriers and facilitators to healthcare access

The second research question examined barriers and facilitators to healthcare access. The evidence shows that access was shaped by six interrelated dimensions: health literacy, legal and administrative status, financial resources, discrimination and trust, language and communication, and geographical mobility.

Health literacy influenced whether families could identify health needs, understand available services, and navigate unfamiliar healthcare systems. Migrant families often had limited knowledge of available services and host-country healthcare systems (Alshamary et al., 2024; Charania et al., 2020). Traditional health beliefs, preferences for home remedies or alternative medicine, dissatisfaction with provider communication, and doubts about prescribed medications could also delay professional consultation and timely intervention (Alshamary et al., 2024; Committee on Community Health Services, 2005; Fernandes et al., 2015; Kovar et al., 2021). Among left-behind children, care-seeking often depended on grandparents or other caregivers who may themselves have limited health literacy or difficulty navigating healthcare systems (Fellmeth et al., 2018; Guan et al., 2018; Hossain et al., 2026). More broadly, migrant children participated less frequently in preventive care and health surveillance programmes (Inácio et al., 2025).

Legal and administrative conditions also restricted access. Undocumented or precariously documented children could be excluded from health insurance coverage or face uncertainty in entitlement, with access sometimes dependent on discretionary decisions by healthcare providers (Hacker et al., 2015; Ruiz-Casares et al., 2013). Healthcare entitlements for migrant children varied widely across Europe and Australia despite formal commitments to children’s rights (Østergaard et al., 2017; Palm et al., 2017). Fear of deportation or other legal consequences could discourage undocumented families from contacting services, even where urgent care was technically available (Alshamary et al., 2024; Committee on Community Health Services, 2005). In Türkiye, registered refugees under temporary protection were entitled to free healthcare, whereas unregistered refugees faced greater restrictions and were often limited to emergency care (Baris et al., 2022). Children with chronic diseases also faced administrative barriers and would benefit from coordinated international strategies for early detection and long-term care (Yoldaş Çelik and Köşeci, 2026). Further barriers included difficulty obtaining healthcare user numbers, delays in registering with primary care providers, long waiting times, disrupted follow-up, problems obtaining prescribed medication, shortages of health personnel, and limited specialised resources (Alshamary et al., 2024; Ibrahim et al., 2003; Inácio et al., 2025; Kovar et al., 2021).

Financial barriers were another major determinant of access. Costs associated with consultations, medication, diagnostic procedures, transport, and time away from work created substantial burdens for families (Alshamary et al., 2024; Ambika et al., 2026; Ayas et al., 2022; Baris et al., 2022; Connor et al., 2007; Hossain et al., 2026; Inácio et al., 2025; Kemei et al., 2023; Mostafa et al., 2021). In some contexts, the absence of a definitive health user number limited state reimbursement for consultations (Inácio et al., 2025). Families therefore sometimes postponed or forwent care, particularly when out-of-pocket expenses were high (Baris et al., 2022). Financial difficulties were also linked to unstable employment, unemployment, services not covered by universal health plans, poverty, housing insecurity, and food insecurity (Alshamary et al., 2024). Among left-behind children, financially motivated maternal migration was associated with higher odds of wasting, stunting, and underweight (Hossain et al., 2026).

Discrimination, stigma, and distrust affected the acceptability of services. Cultural differences, experiences of discrimination, limited cultural competence among professionals, and stigma associated with migrant status, mental health problems, or specific medical conditions could discourage families from seeking or continuing care (Baris et al., 2022; Rosenberg et al., 2022; Ruiz-Casares et al., 2013). The absence of targeted programmes and migrant-sensitive healthcare policies meant that services often failed to respond adequately to children’s social, cultural, and developmental needs (Baris et al., 2022; Rosenberg et al., 2022). Fayad et al. (2024) similarly identified stigma and discrimination among refugee children with disabilities in Jordan, where children were often excluded from social programmes supporting healthy development and learning.

Language and communication barriers recurred across settings. Lack of interpreters, limited availability of bilingual professionals, unfamiliar medical terminology, and inadequate culturally responsive communication affected families’ ability to understand health needs, book appointments, consent to treatment, follow instructions, and trust providers (Alshamary et al., 2024; Ayas et al., 2022; Karim et al., 2020; Kemei et al., 2023; Srichampa et al., 2019). These barriers could contribute to misunderstandings, misdiagnoses, and inadequate treatment (Baris et al., 2022). Research with Congolese refugee children in South Africa showed that language barriers, discrimination, and poor provider communication restricted healthcare utilisation even where services were formally available, demonstrating that service presence alone does not ensure effective access (Meyer-Weitz et al., 2018). Children in conflict-affected settings, including Yemen, also faced language and cultural barriers when accessing healthcare (Hossain et al., 2026).

Geographical mobility and service location further limited access. Children in transit, migrant farmworker children, and mobile families had difficulty maintaining records, completing vaccination schedules, and attending follow-up appointments (Ambika et al., 2026; Connor et al., 2007; Naranjo et al., 2023). Migrant and left-behind children often lived in rural, remote, or underserved areas where healthcare facilities and specialised services were limited (Mostafa et al., 2021). Long travel distances, inadequate transportation, and the time and costs associated with attending appointments could prevent timely care (Baris et al., 2022; Inácio et al., 2025; Kemei et al., 2023). In conflict-affected and displaced settings, services were often insufficiently resourced, particularly for mental health, infectious disease care, immunisation, and primary care (Ibrahim et al., 2003). Refugee and asylum-seeking children could also experience disrupted care during reception, relocation, or transitions between services (Barghadouch et al., 2019; Sandahl et al., 2013). Former unaccompanied immigrant minors in the United States similarly encountered difficulties maintaining continuity of care when moving from shelter-based healthcare to community services (Misra et al., 2022). These barriers frequently overlapped, with migrant families reporting bureaucratic, economic, geographical, and language-related difficulties and often using emergency departments as entry points into care rather than planned routes (Inácio et al., 2025).

The literature also identified several facilitators that can strengthen access. At the individual and community level, access improved when families knew about available services and understood how to navigate them. Community clinics, outreach programmes, refugee health services, and community-based models provided visible and trusted entry points into care (Connor et al., 2007). Routine health assessments, including post-arrival check-ups, supported early identification of needs while introducing families to available services (Kovar et al., 2021). Health education, stronger links between education and health systems, and support in navigating levels of care further improved families’ ability to use healthcare effectively (Fernandes et al., 2015; Karim et al., 2020; Rosenberg et al., 2022). Parental education and mothers’ use of healthcare services were also associated with a higher likelihood of full child immunisation (Hu et al., 2013).

Cultural mediation and trust-building facilitated engagement with care. Trust, cultural understanding, and the ability to reconcile healthcare beliefs from countries of origin with those of the host country were central to healthcare utilisation (Karim et al., 2020). Interpreters trained in multicultural sensitivity, bilingual healthcare workers, volunteers, social networks, community organisations, religious groups, and culturally responsive services helped reduce cultural and linguistic barriers and strengthen relationships between families and providers (Ayas et al., 2022; Baris et al., 2022; Meyer-Weitz et al., 2018; Srichampa et al., 2019; Yoldaş Çelik and Köşeci, 2026). Outreach, benefit and assistance programmes, cultural training, educational services, and workshops were also identified as important facilitators (Alshamary et al., 2024). In mental health provision, mindfulness-based interventions were described as trauma-informed, accessible, and appropriate for refugee children (Fayad et al., 2024).

Health workforce and institutional support also mattered. Paediatricians and nurses helped families navigate systems, access community resources, receive vaccination support, and benefit from health education, counselling, and professional training initiatives (Committee on Community Health Services, 2005; Kovar et al., 2021). International organisations such as WHO and UNICEF supported immunisation coverage and primary care for children affected by war and displacement, while NGOs provided information, direct assistance, advocacy, educational programmes, and community development initiatives (Ibrahim et al., 2003; Inácio et al., 2025). Outreach-oriented models, including professionals reaching children in their living environments, postnatal visits, newborn screening, mobile clinics, and migrant liaison officers, improved access and continuity of care (Ambika et al., 2026; Connor et al., 2007; Kusuma et al., 2010; Yoldaş Çelik and Köşeci, 2026). Schools also supported identification, referral, counselling, school-based healthcare, and vision screening (Guan et al., 2018; Inácio et al., 2025; Misra et al., 2022).

Financial protection facilitated access by reducing out-of-pocket costs. Free healthcare schemes, healthcare vouchers, subsidised medication, humanitarian assistance, and other forms of support helped families obtain necessary care (Baris et al., 2022; Guan et al., 2018; Kemei et al., 2023; Mostafa et al., 2021; Poyraz Fındık et al., 2021). Vouchers for free eyeglasses, for example, significantly increased uptake and use of vision correction among children (Guan et al., 2018). Community organisations, charities, religious groups, UNICEF, WHO, and NGOs also provided financial, logistical, and emotional support (Ibrahim et al., 2003; Kemei et al., 2023; Mostafa et al., 2021). Stable household employment increased the likelihood of full immunisation and healthcare utilisation (Kusuma et al., 2010), while greater maternal financial autonomy was associated with lower rates of child stunting (Hossain et al., 2026).

Finally, inclusive policy frameworks were central to equitable access. Government initiatives that expanded healthcare coverage and enabled free access to services helped reduce disparities (Baris et al., 2022; Guan et al., 2018). Policies ensuring parity of access with residents, government financing of health examinations, and guaranteed access to preventive care, vaccinations, primary care, and specialist services supported timely and equitable provision (Sandahl et al., 2013). Systematic health assessments on arrival, mental health screening, interpreter services, culturally appropriate materials, and other practical supports helped ensure that formal rights translated into usable services (Baris et al., 2022; Karim et al., 2020; Meyer-Weitz et al., 2018; Sandahl et al., 2013). Health literacy interventions, multilingual information on rights and services, workshops for newly arrived migrants, school-based identification and referral pathways, streamlined administrative procedures, protection during asylum processes, and active engagement of migrant families in service planning further improved access to care (Akhtar et al., 2022; Barghadouch et al., 2016).

### 3.4 Gaps in service provision

The third research question examined gaps in healthcare service provision. Six gaps were especially prominent across the literature.

First, preventive and primary care were often weaker than emergency or crisis-based provision. The absence of robust primary healthcare initiatives contributed to overreliance on emergency services, which are more costly and less effective in supporting long-term health outcomes (Markkula et al., 2018; Mostafa et al., 2021; Rosenberg et al., 2022). Many migrant families lacked awareness of the importance of primary healthcare or faced barriers to accessing it, limiting opportunities for early intervention and prevention (Connor et al., 2007; Dutta et al., 2017; Inácio et al., 2025). As a result, children often entered healthcare systems only once conditions had become acute, reflecting missed opportunities for earlier identification, prevention, and follow-up (Baris et al., 2022; Guan et al., 2018; Hjern et al., 1991; Markkula et al., 2018). Gaps in immunisation education and promotion also left children vulnerable to vaccine-preventable infectious diseases (Charania et al., 2020; Fernandes et al., 2015; Hu et al., 2013; Ibrahim et al., 2003; Kovar et al., 2021; Kusuma et al., 2010; Srichampa et al., 2019). In addition, children with chronic conditions often lacked continuity of care, while poor access to dental services contributed to untreated oral health problems (Connor et al., 2007; Hjern et al., 1991; Markkula et al., 2018).

Second, mental healthcare was insufficiently integrated into child migrant health provision. Although trauma, anxiety, depression, behavioural concerns, and emotional distress were recurrently identified across the evidence base, access to child and adolescent mental health services was often delayed, fragmented, underused, or culturally mismatched (Barghadouch et al., 2016; Inácio et al., 2025; Poyraz Fındık et al., 2021; Rosenberg et al., 2022). Prolonged screening and assessment processes could delay the identification of mental and behavioural health needs (Mostafa et al., 2021), while shortages of specialised mental health resources constrained care for children coping with trauma, anxiety, depression, and displacement-related stress (Pesantes and Documet, 2017). Comparative research from Nordic countries similarly showed that health systems remained predominantly focused on somatic care, with less attention to mental health, family support, child participation, and health-promoting environments (Barghadouch et al., 2019).

Third, evidence on left-behind children’s access to healthcare remained limited. Existing studies identified risks associated with parental migration, caregiving arrangements, nutrition, mental health, and health-seeking behaviour (Fellmeth et al., 2018; Hossain et al., 2026), but there was little comparative evidence on their access to preventive care, chronic care, mental health services, or health-system navigation. Available evidence also suggested that these gaps may be compounded by weak referral pathways, as healthcare providers did not always have sufficient knowledge of local community resources or support systems (Ayas et al., 2022; Baris et al., 2022).

Fourth, comprehensive and family-centred healthcare programmes tailored to migrant communities were often absent. Services were not always adapted to the cultural, linguistic, and situational needs of migrant children and families, leaving developmental delays and broader psychosocial needs inadequately addressed (Connor et al., 2007; Committee on Community Health Services, 2005; Hu et al., 2013; Srichampa et al., 2019). In addition, inconsistencies between healthcare professionals’ stated commitment to human rights and their attitudes towards providing care for undocumented children raised concerns about inequitable and discretionary service provision (Connor et al., 2007; Murphy et al., 2020; Ruiz-Casares et al., 2013).

Fifth, data systems and accountability mechanisms were weak. Few studies provided disaggregated evidence by legal status, age, gender, disability, migration pathway, time since arrival, or caregiving arrangement. Children’s own perspectives were also under-represented, despite evidence that children can provide meaningful accounts of health, illness, and care when appropriate methods are used (Fernandes et al., 2015). These gaps limit the ability of policymakers and practitioners to design services that respond to children’s lived realities rather than broad adult-defined categories.

Sixth, cross-border coordination was underdeveloped. Displaced and migrant children are often embedded in transnational social fields, connected to different health understandings, practices, and systems across national borders. Health services therefore need stronger coordination across countries, health authorities, humanitarian organisations, and international agencies to improve early screening, detection, treatment continuity, and long-term care across children’s development and life course (Yoldaş Çelik and Köşeci, 2026).

## 4 Discussion

### 4.1 From formal availability to meaningful access

This review shows that healthcare services for migrant and left-behind children cannot be understood only in terms of formal availability. Across the evidence base, services often existed in principle but were difficult to use in practice because of the barriers mapped in the Results section. Levesque et al.’s (2013) framework is useful for interpreting these findings because it conceptualises access as a multidimensional process shaped by the interaction between service characteristics and the capabilities of individuals and families. The framework identifies five dimensions of services: approachability, acceptability, availability and accommodation, affordability, and appropriateness, alongside five corresponding abilities of populations: the ability to perceive, seek, reach, pay, and engage with healthcare (Cu et al., 2021).

Viewed through this framework, the findings suggest that barriers to care do not operate in isolation. Limited information may restrict the ability to recognise need or identify services; language barriers, cultural mismatch, and discrimination may weaken trust and reduce care-seeking; geographical distance and mobility may prevent families from reaching services; direct and indirect costs may make care unaffordable; and fragmented or culturally unresponsive pathways may undermine continuity and engagement. These dimensions are cumulative. Legal exclusion may intensify financial insecurity, poor information may delay care-seeking, and disrupted pathways may prevent follow-up. Improving access therefore requires more than expanding service availability. It requires health systems that address the legal, social, economic, cultural, and organisational conditions that shape whether children can actually receive timely and appropriate care.

### 4.2 A fragmented ecology of care

The review also reveals a fragmented ecology of care. Public services, schools, NGOs, community organisations, churches, humanitarian programmes, and informal networks all appear in the literature as part of the healthcare access landscape. This plurality can be protective when services are coordinated, trusted, and culturally responsive. However, it can also become inequitable when families must rely on temporary projects, charitable provision, or professional goodwill to obtain care that should be guaranteed as a child right (Murphy et al., 2020; United Nations, 1989).

For left-behind children, this ecology of care is further complicated by parental absence and substitute caregiving. Healthcare decisions may be mediated by grandparents, relatives, or community members, while parents remain involved emotionally or financially from a distance (Guan et al., 2018). Healthcare access for these children should therefore be understood as a relational process involving children, caregivers, transnational parents, schools, local services, and, in some cases, cross-border communication and remittances.

### 4.3 Heterogeneity and the limits of one-size-fits-all policy

Migrant and left-behind children are often grouped together as vulnerable populations, but this review shows that vulnerability is not uniform. Refugee children, asylum-seeking children, undocumented children, unaccompanied minors, children in transit, migrant farmworker children, internal migrant children, and left-behind children differ in their legal statuses, care arrangements, health risks, and routes into services. Policies that treat all migrant children as a single category risk obscuring important differences in need, entitlement, and access.

At the same time, the review identifies shared structural barriers across groups and settings, particularly those related to entitlement, documentation, affordability, language, trust, mobility, and continuity of care. The implication is not that all groups require the same intervention, but that child healthcare systems should combine universal entitlements with targeted supports that recognise differentiated risks and circumstances.

## 5 Implications for policy, practice, and research

National and local health systems should guarantee access to essential healthcare for all children, regardless of migration status. This includes primary care, vaccination, emergency care, maternal and child health services, developmental screening, vision screening, dental care, mental healthcare, and specialist referral where clinically indicated. Rights-based commitments need to be translated into clear eligibility rules, professional guidance, and accountability mechanisms so that access does not depend on discretionary interpretation at the point of care.

Preventive and primary care should be strengthened by connecting children affected by migration early to registration, catch-up immunisation, developmental screening, nutritional assessment, dental and vision checks, and pathways for chronic or specialist care. These pathways should not depend on secure documentation, stable residence, or adult-level health-system literacy.

Mental healthcare should be integrated into migrant and refugee child health provision through trauma-informed, culturally responsive, and developmentally appropriate support linked to primary care, schools, community organisations, and child protection systems. Services should also be linguistically and culturally accessible. Interpreters, bilingual professionals, translated materials, culturally trained staff, and health-navigation support are not optional additions; they are central to safe and equitable care.

Community-based and school-linked models should be expanded where they improve reach, trust, and continuity. However, these models should remain connected to statutory health systems so that NGOs, schools, and communities are not left to compensate for gaps in state provision.

Finally, health systems need stronger data and accountability. Disaggregated monitoring by age, gender, legal status, migration pathway, disability, location, and caregiving arrangement would support more precise planning. Children’s perspectives should also be included through ethical, age-appropriate, and culturally sensitive methods, particularly in research and service evaluation.

Future research should prioritise four areas. First, more empirical studies are needed on left-behind children’s healthcare needs and service access, including preventive care, mental health, chronic care, nutrition, caregiving arrangements, and healthcare decision-making. Second, more evidence is needed from low- and middle-income countries, countries of origin, transit contexts, and regions experiencing large-scale displacement or labour migration. Third, studies should move beyond documenting barriers to evaluating interventions. Promising approaches, including interpreters, outreach, community partnerships, school-linked services, health-navigation support, and inclusive insurance schemes, require stronger evidence on effectiveness, cost, acceptability, and scalability. Fourth, children’s own perspectives should be more systematically included, especially among younger children.

## 6 Strengths and limitations

A key strength of this review is that it brings together dispersed evidence on healthcare services for children directly and indirectly affected by migration. By including migrant, refugee, asylum-seeking, undocumented, internally displaced, unaccompanied, in-transit, and left-behind children, the review identifies both shared barriers and population-specific concerns. It also adopts a broad understanding of access, extending beyond service availability to include entitlement, affordability, language, trust, cultural responsiveness, and continuity of care.

The review also has limitations. Searches were limited to Web of Science/MEDLINE and Scopus, and to English-language peer-reviewed publications, which may have excluded relevant studies from specialist biomedical databases, regional databases, grey literature, and non-English sources. The search strategy prioritised healthcare service terms, meaning that studies indexed primarily under access, utilisation, barriers, or entitlement terminology may have been missed. The included publications were heterogeneous in design, population, and service focus, limiting comparability. Consistent with scoping review methodology, no formal quality appraisal was undertaken; the review therefore maps the evidence rather than assessing the strength of individual findings. The protocol was not formally registered, and the available indexed literature remains geographically uneven, likely under-representing locally produced knowledge from countries of origin, transit settings, and parts of the Global South.

## 7 Conclusion

Migrant and left-behind children require healthcare systems that recognise both their universal rights as children and the specific vulnerabilities created by migration, displacement, legal precarity, and family separation. This review shows that their health needs are broad and interconnected, but that access to care is often constrained by legal, financial, linguistic, geographical, cultural, and organisational barriers.

The findings suggest that healthcare inequity is not only a matter of service scarcity. It also reflects systems that remain insufficiently child-centred, culturally responsive, legally inclusive, and attentive to the realities of transnational and disrupted family lives. When services are difficult to understand, reach, afford, trust, or sustain over time, formal availability does not become meaningful access.

Strengthening healthcare for migrant and left-behind children therefore requires universal child healthcare entitlements, robust preventive and primary care pathways, integrated mental health support, linguistically and culturally accessible services, community and school-linked access points, and stronger cross-sector and cross-border coordination. Above all, migrant and left-behind children should not be treated as marginal or exceptional users of healthcare systems, but as children whose rights, needs, and lived experiences must be built into mainstream health policy and practice.

## Ethical standards statement

All authors have read, understood, and have complied, as applicable, with the statement on ‘Ethical responsibilities of Authors’ as found in the Instructions for Authors.

### Data availability

All data used in this review were extracted from published studies. The extracted study characteristics are provided in the appendix.

## CRediT authorship contribution statement

**Jane Spiteri:** Writing – review & editing, Writing – original draft, Supervision, Methodology, Formal analysis, Data curation, Conceptualization. **Anne Carolina Ramos:** Writing – review & editing, Writing – original draft, Formal analysis, Data curation. **Catarina Pinheiro Mota:** Writing – original draft, Formal analysis, Data curation. **Egemen İpek:** Writing – original draft, Methodology, Formal analysis, Data curation. **Viorela Ducu:** Writing – original draft, Methodology, Formal analysis, Data curation. **Bárbara Bäckström:** Writing – review & editing, Writing – original draft, Formal analysis, Data curation.

## Declaration of competing interest

The authors declare that they have no known financial interests or personal relationships that could have appeared to influence the work reported in this paper.

## Appendix Supplementary materials

Image, application 1
