# Supplementary material for: Access to healthcare services for migrant and left-behind children: A scoping review
Source: J Migr Health. 2026 Jul 1;14:100425. doi: 10.1016/j.jmh.2026.100425 (PMC13377135; doi:10.1016/j.jmh.2026.100425)
Supplement: Supplementary file 1 [file mmc1.docx]

**Appendix 1. Supporting information**

| **Author & Year** | **Country** | **Title** | **Document Type** | **Population** | **Healthcare Service** | **Design** | **Key Findings** |
| --- | --- | --- | --- | --- | --- | --- | --- |
| Hjern et al. (1991) | Sweden | Health and nutrition in newly resettled refugee children from Chile and the middle-east | Article | Newly resettled refugee children from Chile and the Middle East | Paediatric assessment, nutrition assessment, dental and child health follow-up | Paediatric outpatient assessment | Refugee children required nutritional, dental and chronic-condition follow-up. Resettlement enabled access to healthcare, but continuous care remained necessary. |
| Ibrahim et al. (2003) | South-eastern Europe | Decline in the child health indicators: assessing the impact of war in five south-eastern European countries | Article | Children and families affected by war and displacement | General healthcare and mental healthcare | Literature / Medline review | War and displacement were associated with worsening child health indicators, including mortality, malnutrition, infectious diseases, poor immunisation coverage and mental health needs. Health systems were inadequately resourced. |
| Committee on Community Health Services (2005) | United States | Providing care for immigrant, homeless, and migrant children | Review | Immigrant, homeless and migrant farmworker children | Paediatric care, screening, immunisation, dental care, mental health support | Clinical policy/guidance statement | Children required comprehensive paediatric care addressing infectious disease, immunisation, dental problems, mental health and acculturation stress. A medical-home model was recommended. |
| Connor et al. (2007) | United States | Increasing the delivery of health care services to migrant farm worker families through a community partnership model | Article | Children of migrant farmworker families | Physical examinations, screenings, health education, referrals | Action research/ community partnership model | Community partnership improved delivery of healthcare to mobile migrant families. Isolation, poverty, frequent mobility, language barriers and low literacy impeded access. |
| Kusuma et al. (2010) | India | Migration and immunization: Determinants of childhood immunization uptake among socioeconomically disadvantaged migrants in Delhi, India | Article | Socio-economically disadvantaged rural–urban migrant children under two | Childhood immunisation | Cross-sectional survey | Immunisation uptake was lower among migrant children, particularly recent migrants. Maternal age, maternal education, healthcare use and secure employment improved uptake. |
| Ruiz-Casares et al. (2013) | Canada | Access to health care for undocumented migrant children and pregnant women: The paradox between values and attitudes of health care professionals | Article | Undocumented migrant/refugee children and pregnant women in Quebec | General healthcare services | Online expert survey | Access was constrained by exclusion from universal coverage and discriminatory or inconsistent professional attitudes. The study highlighted a gap between human-rights values and actual access for undocumented children. |
| Hu et al. (2013) | China | Decline in the child health indicators: assessing the impact of war in five south-eastern European countries | Article | Migrant children under two and their mothers | Childhood immunisation | Cross-sectional survey | Migrant children, especially recent migrants, had lower age-appropriate immunisation coverage. Parental education, household income and mothers’ use of healthcare increased immunisation uptake. |
| Sandahl et al. (2013) | Nordic countries: Denmark, Iceland, Norway and Sweden | Policies of access to healthcare services for accompanied asylum-seeking children in the Nordic countries | Article | Accompanied asylum-seeking children under 18 | Primary care, preventive care, health examinations, vaccination and some specialist care | Comparative policy analysis | Entitlements varied across Nordic countries. Gaps included inconsistent health examinations, uneven mental health screening and limited data on actual service quality. |
| Fernandes et al. (2015) | Australia | What makes people sick? Burmese refugee children's perceptions of health and illness | Article | Burmese refugee children | Doctors, hospitals and medicines | Qualitative child-centred study using interviews and drawings | Children understood health through environmental, dietary and biomedical explanations. Language limitations and acculturation shaped healthcare understanding and use. |
| Barghadouch et al. (2016) | Denmark | Refugee children have fewer contacts to psychiatric healthcare services: an analysis of a subset of refugee children compared to Danish-born peers | Article | Refugee children under 18 with residence in Denmark | Psychiatric hospital, outpatient, emergency and private psychiatric/psychological care | Nationwide registry cohort study | Refugee children had fewer psychiatric healthcare contacts than Danish-born peers despite likely mental health need. This suggested under-recognition, referral gaps and access barriers. |
| Dutta et al. (2017) | India | Primary immunization coverage among migrant children in the age group of 12 to 23 months in Sriperumbudur Taluk, Kanchipuram District | Article | Migrant children aged 12–23 months in construction-site communities | Primary immunisation | Community-based cross-sectional descriptive study | Immunisation coverage was high in the study setting, but lack of time was the reason for partial immunisation. Health education for migrant workers was recommended. |
| Guan et al. (2018) | China | Health seeking behavior among rural left-behind children: Evidence from Shaanxi and Gansu provinces in China | Article | left-behind children in China, whose parents have migrated to urban areas for work, leaving them behind in rural areas, under the care of elderly grandparents or other relatives. The study also includes non-left-behind children for comparison. | vision care, distribution of eyeglasses. vision screenings and the provision of eyeglasses through a voucher system that allowed children to redeem a free pair of prescription glasses at designated optical stores. | a randomized controlled trial involving 13,100 students from grades 4 and 5 in rural primary schools. Vision health care services were provided as part of the intervention, and data were collected through surveys and vision examinations. | Providing vouchers for free eyeglasses significantly increased the uptake and usage of eyeglasses among children. Also, Conducting vision screenings at schools helped identify children in need of vision correction and facilitated access to care. The vouchers, implementing subsidy programs, providing free eyeglasses, can significantly improve healthcare access (more than distance or an health problem, financial barriers were identified). this study used eye/vision care, but research is needed to explore other health issues and services required by left-behind children. |
| Meyer-Weitz et al. (2018) | South Africa | Healthcare service delivery to refugee children from the Democratic Republic of Congo living in Durban, South Africa: a caregivers' perspective | Article | Congolese refugee children aged 0–10 and caregivers | Public clinics, hospitals, immunisation and child health consultations | Explanatory mixed-methods study | Free public healthcare facilitated access, but language barriers, waiting times, poverty, discrimination and lack of information constrained care. Social networks and NGOs supported navigation. |
| Markkula et al. (2018) | Mainly Europe and North America | Use of health services among international migrant children - a systematic review | Review | International migrant children under 18 | Primary, preventive, dental, mental health, emergency and hospital care | Systematic review | Migrant children used preventive, primary and dental care less, but emergency and hospital care more. Major gaps included limited evidence from Asia, Africa and South America. |
| Srichampa et al. (2019) | Thailand, Malaysia and Singapore | Managing unskilled migrant labour: Language diversity in multicultural Southeast Asian societies | Article | Migrant labourers and their families | Language support and communication in service contexts | Language/policy analysis | Language diversity was a major access issue for migrant labour populations. Retain only if the review includes family-level communication barriers affecting children’s healthcare. |
| Barghadouch et al. (2019) | Denmark, Finland, Norway and Sweden | Do health reception policies in the Nordic region recognize the rights of asylum-seeking and resettled refugee children? | Article | Asylum-seeking and resettled refugee children | Health reception policies, screening, vaccination, oral health, primary care and mental health | Comparative policy analysis | Policies often prioritised communicable disease screening over holistic child health. Mental health, participation, family support and child-rights implementation were uneven. |
| Charania et al. (2020) | New Zealand | Vaccine-Preventable Disease-Associated Hospitalisations Among Migrant and Non-migrant Children in New Zealand | Article | Migrant and non-migrant children from birth to age five | Vaccination and hospital care for vaccine-preventable diseases | Retrospective population cohort study | Hospitalisation for vaccine-preventable diseases varied by migrant background and visa category. Refugee-background children experienced disproportionate burdens. |
| Karim et al. (2020) | UK, Sweden, United States and Australia | Immigrant parents' experiences of accessing child healthcare services in a host country: A qualitative thematic synthesis | Review | Voluntary immigrant parents accessing child healthcare | Primary care, developmental surveillance, vaccination, specialist referrals and child health clinics | Qualitative thematic synthesis | Parents struggled with navigation, language, cultural differences, waiting times and mistrust. Interpreters, respectful communication and community networks facilitated access. |
| Mostafa et al. (2021) | Lebanon | Congenital Heart Disease in Syrian Refugee Children: The Experience at a Tertiary Care Center in a Developing Country | Article | Syrian refugee children under 18 referred for cardiac evaluation | Tertiary cardiac care, surgery, catheterisation, follow-up | Retrospective medical record review | Syrian refugee children presented late with congenital heart disease and had higher surgical mortality than non-Syrian children. Financial, geographical and specialist-care barriers delayed diagnosis and treatment. |
| Kovar et al. (2021) | United States | HPV vaccine promotion: Snapshot of two health departments during the COVID-19 pandemic | Article | Adolescents aged 11–18, including insured and uninsured adolescents | HPV vaccination, sexual health screening and treatment | Practice snapshot/literature-informed analysis | COVID-19 disrupted routine vaccination. Education, counselling and opportunistic vaccination during clinical encounters were identified as facilitators. Retain only if adolescent preventive care is within scope. |
| Poyraz Fındık et al. (2021) | Turkey | Mental health need and psychiatric service utilization patterns of refugee children in Turkey: A comparative study | Article | Syrian refugee children and Turkish comparison group in psychiatric care | Specialised child and adolescent mental healthcare | Comparative clinical study | Refugee children had high trauma exposure, depression, PTSD and comorbidity, yet attended fewer follow-up appointments. Free entitlement did not ensure continuity of mental healthcare. |
| Ayas et al. (2022) | Turkey | Development of preschool refugee children living under temporary protection status | Article | Syrian preschool refugee children under temporary protection | Primary healthcare; developmental screening | Case-control developmental study | Developmental delay was more frequent among refugee children than Turkish controls. Language barriers and economic hardship limited access, while interpreters supported communication. |
| Baris et al. (2022) | Turkey | Rates of emergency room visits and hospitalizations among refugee and resident children in a tertiary hospital in Turkey | Article | Syrian refugee and resident children attending a tertiary hospital | Emergency department and inpatient hospital care | Retrospective observational study | Refugee children had higher emergency visits and hospitalisations, especially among neonates and infants. Findings suggest insufficient access to preventive and primary care. |
| Rosenberg et al. (2022) | United States | Recently-Arrived Afghan Refugee Parents’ Perspectives About Parenting, Education and Pediatric Medical and Mental Health Care Services | Article | Recently arrived Afghan refugee families | Preventive paediatric care and mental healthcare | CBPR qualitative study using interviews | Parents valued preventive care but reported delays in recognising and addressing children’s mental and behavioural health needs. Language, stigma and adjustment stress shaped access. |
| Misra et al. (2022) | United States | Systematic review of former unaccompanied immigrant minors’ access to healthcare services in the United States | Review | Former unaccompanied immigrant minors | Medical, dental, mental health, screening, vaccination, counselling and community services | Systematic review | Initial services were often available in shelters, schools or foster care, but continuity after release was weak. Insurance, legal status, language, transport and referral barriers limited access. |
| Akhtar et al. (2022) | New Zealand | Access to the healthcare system: Experiences and perspectives of Pakistani immigrant mothers in New Zealand | Article | Pakistani immigrant mothers with children under 12 | Free GP care, vaccination, screening and emergency care | Qualitative semi-structured interviews | Mothers reported limited system knowledge, transport difficulties, long waits, rushed appointments and delayed GP access. Education, experience and after-hours/emergency services facilitated care. |
| Kemei et al. (2023) | Ethiopia | The forms and adverse effects of insecurities among internally displaced children in Ethiopia | Article | Internally displaced children (IDPs) living in the Burayu IDP camp, Ethiopia, together with their parents/guardians and service providers. The study included 20 children (12–15 years), 20 parents/guardians, and 13 service providers. | Healthcare was initially provided through a camp clinic, health centres, hospitals, and health extension workers. Services included primary healthcare, referrals, medications, maternal and child healthcare, health education, and nutritional support. | Exploratory qualitative case study guided by an intersectionality framework. | .Internally displaced children experienced multiple interconnected insecurities affecting their well-being: (1) basic needs insecurity (shelter, clothing, water, sanitation), (2) healthcare insecurity (financial barriers, lack of drugs, poor-quality care), (3) academic insecurity (school disruption, discrimination, language barriers), (4) economic insecurity (loss of income and employment), (5) food insecurity (hunger, malnutrition risks), and (6) physical and mental health insecurity (violence, trauma, fear, risk of PTSD and depression). The study concludes that socioeconomic and contextual factors intersect to negatively affect children's health and well-being and that coordinated, multi-sector interventions are needed. |
| Naranjo et al. (2023) | Darién Gap/Latin American transit corridor | The Endless Vulnerability of Migrant Children In-Transit across the Darien Gap | Review | Migrant children in transit, including unaccompanied minors and infants | Emergency care, screening, vaccination, nutrition, mental health and maternal/newborn care | Narrative literature review | Transit exposed children to malnutrition, infectious disease, incomplete immunisation, violence, exploitation and trauma. Mobility, lack of documents and weak in-transit services limited continuity of care. |
| Alshamary et al. (2024) | Canada | Barriers and facilitators to health care access for migrant children in Canada: A scoping review | Review | Migrant children | Healthcare access broadly, including primary, preventive and specialist services | Scoping review | The review mapped barriers and facilitators to healthcare access for migrant children in Canada, highlighting systemic, financial, language, communication and navigation barriers. |
| Fayad et al. (2024) | Jordan | Refugee Caregivers’ Perceptions of Using Mindfulness-Based Interventions to Support Coping Skills in Children with Disability in Jordan | Article | Refugee caregivers of children with disabilities | Occupational therapy-related support; mindfulness-based mental health interventions | Open-ended caregiver survey with descriptive coding | Children faced behavioural, emotional and cognitive challenges. Financial, environmental, logistical and stigma-related barriers limited support. Caregivers viewed mindfulness-based interventions positively, but access barriers remained. |
| Calderon et al. (2024) | Latin America and the Caribbean | Health conditions of migrant children and adolescents from Latin America and the Caribbean: A narrative review; [Salud de niños, niñas y adolescentes migrantes de América Latina y El Caribe: revisión narrativa de literatura] | Review | Migrant children and adolescents under 18 | Emergency, primary, outpatient, preventive, mental, sexual/reproductive, vaccination and dental care | Narrative review | Children faced infectious diseases, malnutrition, oral health problems, mental health concerns, violence, pregnancy-related risks and incomplete vaccination. Services were often inaccessible or culturally inadequate. |
| Inácio et al. (2025) | Portugal | Access and utilization of Portuguese mental healthcare services by migrant children and adolescents: perceptions and experiences of families | Article | Migrant children, adolescents and families | Mental healthcare services | Qualitative exploratory focus groups | Families faced bureaucratic, economic, geographical and language barriers. Emergency departments were often used as entry points; NGOs, schools, informal networks and translation tools facilitated access. |
| Ambika et al. (2026) | India, Kerala | Access to Health Care for Under-five Children of Migrant Laborer Settlements in Ernakulam District, Kerala, Southern India: A Mixed Method Study | Article | Under-five children in migrant labourer settlements | Primary healthcare, modern medicine, child health treatment | Mixed-methods study: cross-sectional survey and stakeholder interviews | Migrant children experienced diarrhoea, ARI and wasting. Barriers included health-centre timings, language difficulties and indirect costs. Flexible timings and interpreters were recommended. |
| Yoldaş Çelik and Köşeci (2026) | Türkiye | Refugee children and inherited metabolic disorders: lessons from Türkiye and global implications | Article | Syrian refugee children suspected of inherited metabolic disorders | Diagnosis and long-term specialist care for inherited metabolic disorders | Special/clinical article drawing on Turkish experience | Refugee children faced delayed diagnosis and disrupted long-term care for rare genetic disorders. The study called for globally coordinated early detection and long-term management. |
| Hossain et al. (2026) | Yemen | When mothers move: the impact of maternal migration motives on child health outcomes in Yemen | Article | Children affected by maternal migration | Nutrition programmes and primary healthcare packages | Quantitative analysis of Yemen MICS data using regression models | Economic maternal migration increased malnutrition risks, while marriage-related migration was associated with mixed health outcomes. Male children and poorer households were especially vulnerable. |

**TABLE A1: Search strategy used for database searching in chronological order**
